# Assessment of Quality of Sleep and Daytime Sleepiness in Medical Professionals and Students in a Medical College : A Descriptive Crosssectional Study

**DOI:** 10.31729/jnma.8780

**Published:** 2024-10-31

**Authors:** Sundar Pandey, Aabishkar Parajuli, Deepak Paudel, Deepak Aryal, Anmol Singh Shrestha, Mahesh Raj Ghimire, Kapil Neupane, Dipim Gautam, Bikash Poudel

**Affiliations:** 1 'Department of Internal Medicine, Devdaha Medical College, and Research Institute, Bhaluhi, Devdaha, Rupandehi, Nepal; 2 Devdaha Medical College and Research Institute, Bhaluhi, Devdaha, Rupandehi, Nepal

**Keywords:** *sleep*, *sleep hygiene*, *sleep quality*

## Abstract

**Introduction:**

Poor sleep quality is a global public health issue particularly more prevalent in medical professionals and students. Despite various health and occupational risks, research is still lacking regarding the quality of sleep and its related factors among medical professionals and students in our region. Therefore, our study aimed to estimate the prevalence of poor sleep quality in this population.

**Methods:**

This observational cross-sectional study was conducted among medical professionals and students of Devdaha Medical College from December 2023 to June 2024 after ethical approval from the Institutional Review Board. Data regarding sleep quality and excessive daytime sleepiness were collected using the Pittsburg Sleep Quality Index (PQSI) and Epworth Sleepiness Scale with premade questionnaires in Google Forms.

**Results:**

Among 395 participants, 260 (65.83%) were in the age group 20-25 years and 200 (50.63%) were nursing students. The prevalence of poor sleep quality was 146 (36.96%; 95% CI: 32.19%-42.93%) and the global mean score of PSQI was calculated to be 5.12. Out of the total participants, 255 (64.55%) had excessive daytime sleepiness, 84 (21.26%) participants had one or more sleep problems out of which, snoring was in 40 (10.12%) participants.

**Conclusions:**

About one-third of the study population poor sleep quality and day time sleepiness was reported in more than half of the respondents.

## INTRODUCTION

Medical professionals and students are exposed to various stressful situations like long duration of working hours, study pressure, sleepless nights, and shift duties which impedes their quality of sleep.^1^

Poor sleep quality causes fatigue, exhaustion, irritation and loss of attention causing impairment in communication and coordination among health workers and it leads to decreased concentration, cognitive decline and poor academic performance in students.^23^ So it is critical to investigate the sleep quality and associated factors to improve sleep hygiene, reduce medical errors and improve overall performance of students.

Globally, the prevalence of poor quality among healthcare professionals varies from 18.4 to 96.1%, in Asia, it ranges from 24.4 to 84.6%.^4^ Studies in Nepal have reported a prevalence of 35.4 to 48.03%.^1^ Studies regarding sleep quality and daytime sleepiness among health professionals and students in Nepal are limited. Considering the high prevalence of poor sleep quality and its consequences, it is crucial to determine the quality of sleep among these populations.

Owing to the importance of sleep quality among healthcare providers and medical students and its effects on patient care and safety, individual health, and academic performance, and given the paucity of local data available, we conducted this study to fill the gap and aimed to estimate the prevalence of poor sleep quality and excessive daytime sleepiness in medical professionals and students.

## METHODS

This observational, cross-sectional study was done on medical professionals (Doctors and Nurses) and students (MBBS, Nursing, Health Assistant and Community Medical Assistant) of medical college for a 7-month duration starting from December 1, 2023 to June 30, 2024. The study was conducted after ethical approval from the Institutional Review Board of Devdaha Medical College and Research Institute (Reference number: 27/2023). Data was collected by online invitation using Google Forms with pre-made valid questionnaires using the Pittsburgh Sleep Quality Index (PSQI)^4^ and the Epworth Sleepiness Scale (ESS).^5^ The study included all the participants who gave consent and responded to the questionnaire. In contrast, those refusing to give consent, those with incomplete and incorrect responses, and those who were already diagnosed with cases of psychiatric illnesses and known sleep disorders on regular medications were excluded from the study.

Assuming the prevalence of poor sleep quality of 58%^6^ at a 95% level of confidence and 5% margin of error, the sample size was calculated using the formula:


$$\begin{array}{l} \text{n}={\text{Z}}^{2}\times \frac{\text{p(1}-\text{p)}}{{\text{e}}^{\text{2}}}\\ \;=1.96^{2}\times \frac{0.58\times (1-0.58)}{0.05^{2}}\\ \;=374.32\text{ (Roundup value of 375 was taken)} \end{array}$$

n = Sample size
Z = critical value of normal distribution for the assumed level of confidence
P = Estimated proportion of parameter of interest
  (the prevalence of poor sleep quality in health professionals)^3^ = 58%
e = Assumed percentage for margin of error

In order to incorporate possible attrition and avoid non-response of study participants, a non-response rate of 5% was added to calculate the final sample size which gave the sample size of 393.75. Thus, we enrolled 395 participants who responded completely to our questionnaire.

Demographic data regarding gender, age, profession, body mass index, exercise, and previous sleep or chronic disease diagnoses was collected and an assessment of sleep quality was done with the Pittsburgh Sleeping Quality Index (PSQI) while the Epworth Sleepiness Scale (ESS) was used to identify individuals with excessive daytime sleepiness.

Pittsburgh Sleeping Quality Index (PSQI) is a validated tool used to assess adult sleep quality and pattern. It contains 19 self-rated questions that generate seven component scales (subscales). The seven components used to calculate PSQI were: subjective sleep quality, sleep latency, sleep duration, habitual sleep efficiency, sleep disturbances, use of sleeping medication, and daytime dysfunction. Sleep latency is the time required to fall asleep after going to bed. Sleep efficiency is the amount of time actually spent on sleep. Sleep duration is the amount of actual time spent in an hour. Each component scores 0-3 points, where 0 score indicates no difficulty and a score of 3 indicates severe difficulty. The total scores of the seven components were added together to give one global score ranging from 0-21 points. A score of 0-5 indicates good sleep quality, more than 5 indicates poor sleep quality and more than 10 indicates sleep disorder.^7^

Epworth Sleepiness Scale (ESS) uses eight items to identify excessive daytime sleepiness, that grade the probability of the individual dozing in inappropriate situations. Participants were asked to respond on a 4-point Likert scale for their chance of dozing or falling asleep. The global score ranges from 0 to 24. Scores of 11 or higher need consultation with a sleep specialist to diagnose and treat the cause of sleepiness.^5^

The scoring system is from 0 to 3 in which

* 0 : would never doze,
* 1 : slight chance of dozing,
* 2 : moderate chance of dozing, and
* 3 : high chance of dozing.

Scores >10 points suggest Excessive Daytime Sleepiness and Scores are classified as follows

0-10 = normal range in healthy adults
10-14 = Mild sleepiness
15-17 = Moderate sleepiness
18 or higher = severe daytime sleepiness.^8^

After the data collection, data from each individual was coded and protected in a password-protected computer. The obtained data was analyzed using Statistical Package for Social Sciences (SPSS) version 19. Categorical variables were calculated as frequency and percentage while continuous variables were calculated as Median±Standard Deviation.

## RESULTS

Out of 395 study participants, 315 (79.74%) were females. 260 (65.83%) participants were in the age group 20-25 years and the median age was 22±2.54 years. Among them, 200 (50.63%) were nursing students, 41 (10.37%) participants were overweight, and 85(21.52%) participants used to exercise at least 30 minutes twice a week. Out of total, 146 (36.96%; 95% CI: 32.19%-41.93%) had PQSI score more than 5 suggesting poor quality sleep (Table 1).

**Table 1:** Demographic variables and poor sleep quality among participants (n=395).

Variables	n (%)	Poor Sleep Quality (PQSI>5) n (%)
**Age**		
<20 years	96 (24.31)	37 (38.54)
20-25 years	260 (65.83)	96 (36.96)
26-30 years	35 (8.86)	10 (28.57)
>30 years	4 (1)	3 (75)
**Sex**		
Male	80 (20.25)	23 (28.75)
Female	315 (79.74)	123 (39.04)
**Level of Education**		
MBBS Students	145 (36.71)	52 (35.86)
Health Workers/Medical Professionals	34 (8.61)	14 (41.17)
Nursing Students	200 (50.63)	75 (37.50)
Allied Health Professionals (HA/CMA)	16 (4.11)	5 (31.25)
**BMI**		
Less than 18.5 (Underweight)	9 (2.27)	3(33.33)
18.5 - 24.9 (Healthy Weight)	344 (87.08)	125(36.33)
25 - 29.9 (Overweight)	41 (10.37)	17(41.42)
30 and above (Obesity)	1 (0.25)	1(100)
**Exercise**		
No	310 (78.48)	116 (37.42)
Yes	85 (21.52)	30 (35.29)

On ESS, 255 (64.55%) participants had score more than 10 suggestive of excessive daytime sleepiness. (Table 2).

**Table 2:** Epworth Day time sleepiness scale among participants (n=395).

Grading (Scale Range)	n (%)
Normal (0-10)	140(35.44)
Mild Daytime Sleepiness (11-14)	82(20.75)
Moderate Daytime Sleepiness (15-17)	159(40.25)
Severe Daytime Sleepiness (≥18)	14(3.54)

The global mean score of PQSI was 5.12. On PQSI, 127 (32.15%) had very good sleep quality, 74 (18.73%) had sleep latency less than 15 minutes, 303 (76.71%) had sleep duration of more than 7 hours, 330 (83.54%) had sleep efficiency more than or equal to 85%, 40 (10.12%) never had sleep disturbance, 357 (90.37%) did not use medication for sleep and 184 (46.59%) did not have daytime dysfunction. Out of total, 84 (21.26%) had one or more sleep problems as reported by their partners of which snoring was 40 (10.12%) (Table 3).

**Table 3:** PSQI variables in participants (n=395).

Component	Normal (%)	Mild Disorder (%)	Moderate Disorder (%)	Severe Disorder (%)
Sleep Quality	Very good	Fairly good	Fairly Bad	Very bad
	127 (32.15%)	254 (64.31%)		14 (3.54%)
Sleep Latency	Less than or =15 min	16-30 min	31-60 min	>60 min
	74 (18.73%)	154 (38.98%)	116(29.36%)	51 (12.91%)
Sleep Duration	>7 hours	6-7 hours	5-6 hours	<5 hours
	303 (76.71%)	58(14.68%)	20(5.06%)	14 (3.54%)
Sleep Efficiency	>or= 85%	75-84.9 %	65-74.9%	<65%
	330 (83.54%)	43(10.89%)	7 (1.78%)	15 (3.79%)
Sleep Disturbance	Never	<1/week	1-2/week	3 or >/week
	40 (10.12%)	290 (73.42%)	60 (15.18%)	5 (1.27%)
Use of Sleep	Not/month	<1/week	1/2/week	3/>/week
Medication	357 (90.37%)	19(4.81%)	11 (2.78%)	8 (2.03%)
Daytime	0	1-2	3-4	5-6
Dysfunction	184 (46.59%)	76 (19.24%)	82 (20.75%)	53 (13.42%)
**Sleep Problem as reported by partner**			**Number n (%)**	
None			311 (78.73)	
Snoring			40 (10.12)	
Long pause between breath while sleep			10 (2.54)	
Leg twitching or jerk during sleep			22 (5.56)	
Episodes of disorientation or confusion			3 (0.76)	
Sleep Talking			19 (4.81)	
Sleepwalking			6 (1.52)	

## DISCUSSION

Sleep problem is an important public health issue and can affect individuals of all ages and populations, particularly health science students and professionals, whose academic careers and duties are highly stressful.^9^ The quality of sleep was assessed using the Pittsburgh Sleep Quality Index. Across the continents, Europe reports the highest prevalence of poor sleep quality followed by the Americas, Africa, and Asia.^10^

In our study, the poor sleep quality among the study participants was 36.96%, the finding was similar to a study done by Joseph et al^11^ (38.1%) at a tertiary care center in Bengaluru but less than the study done by Ravi A et al^6^ (58%), Lujian A et al^12^ (70.1%) in Saudi Arabia and Tur CF et al^13^ (80.6%) in Turkey. This low prevalence in our study may be due to the presence of a diverse group of study population, variations in sleep patterns, and non-use of work shift effect on sleep quality study compared to studies conducted in various parts of world.

Sleep quality varies according to gender differences. In our study, there were more female participants, and also the proportion of poor sleep quality was high in females compared to males. This is similar to a study done by Alost MR et al in Jordan^14^ and Fatima Y et al^15^ at the University of Queensland, Australia. The higher prevalence of poor sleep quality in females is attributed to physiological and hormonal influences, traditional gender roles, household responsibilities, and cultural practices.^16^

Quality of sleep varies among different medical professionals and students. In our study, medical professionals had more problems with sleep quality (41.17%) than MBBS and nursing students. The higher prevalence in medical health professionals might be because health professionals are exposed to long and irregular duty hours including night shifts which disrupt their sleep-wake cycle and further high levels of stress and job demands in healthcare also lead to sleep disturbances and insomnia. The findings in our study are less than the prevalence in a study done by Jahrami et al^16^ (55%) and 66% in a study done by Ravi A et al^6^ in Chennai India because our study included medical students along with health professional while other studies only included healthcare professionals.

Body mass index and sleep quality are closely interrelated to each other. Poor sleep is also associated with high body mass index as sleep deprivation has been shown to increase the level of growth hormone-releasing peptide (Ghrelin) and reduced level of leptin thus increasing hunger and food intake promoting overweight and obesity.^17^

A meta-analysis done by Amri S et al^18^ showed that an increase in body mass index increases the risk of sleep disturbances due to obesity-related hypoventilation and apnea thus compromising sleep quality (odds ratio (OR) of association between body mass index (BMI) ≥ 25 and sleep disturbances: 1.33 and the confidence interval (CI) was between 1.16-1.51).^18^ In our study, the quality of sleep was poorer in participants with abnormal body mass index than in healthy weight patients (41.17% vs 36.3%). However, in a study by Tsegay et al^4^ in North Ethiopia 87.18% of people with abnormal body mass index (BMI) had poor sleep quality. This might be because sleep-related disorders like apnea are more common in patients with high BMI.

Regular physical exercise has been shown to improve sleep quality and symptoms of sleep disorders. A systemic review of various studies by Alnawaar M.A et al^19^ identified physical activity was positively associated with sleep quality, which indicates that physical activities improve sleep quality. Also, it modulates sleep quality by releasing melatonin, which promotes the synthesis and release of a hypnotic neurotransmitter GABA.^20^ Those with regular exercise have good-quality sleep, this was observed in various studies done in Brazil, Spain, and China.^9,21,22^ However, in our study, we did not find much difference in sleep quality among those who exercise at least 30 minutes twice a week and those who do not exercise (37.4% vs 35.29%). The differences in our study might be due to the study being done in a young population and the majority of participants having normal BMI and good sleep hygiene and poor account of psychiatric illness, other illnesses, and work schedules in our study.

The majority of participants (64.55%) in our study had excessive daytime sleepiness as per the Epworth Daytime sleeping scale, which is higher than the study done by Ravi A et al^6^ (45.3%) and Alieu I et al^23^ (47%) in India. The reason for the differences in prevalence might be attributed to variations in sample size, lifestyle, socio-cultural, academic culture, the demographic characteristics of the study participants, and differences in environmental factors.

There were few limitations of our study. Firstly, it included medical professionals and students working and studying in a single-center so the extrapolation of the findings to other population and areas is questionable and secondly, because of the use of self-reported data, there is a chance of responder as well as recall bias and even over/underestimation of the prevalence of poor quality of sleep. Because of unequal participation from various groups, population distribution could not be estimated. Other factors affecting sleep quality like coffee consumption, work shifts, and cigarette or alcohol consumption were not considered in this study. This study warrants a multicenter study with more participants in equal distribution from each sector and consideration of other factors that can affect sleep quality.

## CONCLUSIONS

About one third of the participants reported poor sleep quality while more than half reported of day time sleepiness. However, most of the respondents did not have dysfunction during the day time, similarly most of the participant did not reported taking medication for sleep. Snoring was the most common sleep problem reported by the partner.
